# Transcultural Psychiatry Workshop: Sharing Our Similarities and Celebrating Our Differences

**DOI:** 10.1192/bjo.2025.10254

**Published:** 2025-06-20

**Authors:** Khadeeja Ansar, Mahira Syed, Nazish Hashmi, Christiana Elisha-Aboh, Seonaid Beaumont

**Affiliations:** 1 Leeds and York NHS Foundation Trust, Leeds, United Kingdom; 2 Tees Esk and Wear valley NHS Foundation Trust, York, United Kingdom; 3 Sheffield Health and Social Care NHS Foundation Trust, Sheffield, United Kingdom

## Abstract

**Aims:** Transcultural psychiatry is a critical field that examines the influence of cultural factors on mental health, illness, and treatment across diverse societies. It acknowledges that psychiatric symptoms and disorders can manifest distinctly based on cultural contexts, beliefs, and practices.

The Transcultural Psychiatry Workshop was held face to face at the Sheffield Health and Social Care Trust Grand Round in September 2024. The aim was to aid in implementing comprehensive training programmes, leverage workforce diversity, encourage culturally sensitive interventions, share helpful learning, enhance community engagement.

**Methods:** The workshop was 120 minutes and was attended by 104 doctors. The workshop included a specialist panel of experts from experience, carers and professionals, presentation regarding data regarding healthcare experience of ethnic minority groups in the area and case-based learning opportunity. Pre- and post-workshop surveys were administered to assess the effectiveness of the workshop and to inform future planning and educational strategies. They used a 5 point Likert scale for most questions.

**Results:** There were 75 responses received for the pre-workshop questionnaire and 48 for the post-workshop questionnaire. For the question how likely are you to effectively and consistently intervene when you observe discriminatory behaviours in others, there was an increase from 29.33% to 39.58% to be very likely. Regarding awareness of local policy those answering yes went from 28.00% to 97.92%. Whereas 26.67% of respondents felt their knowledge of transcultural psychiatry was below average and 4.00% felt it was far below average, after the workshop 0.00% rated in these two categories. The understanding of potential barriers for people from ethnic backgrounds when accessing healthcare increased in the above average category from 29.33% before to 66.67% after.

**Conclusion:** The feedback received from participants was predominantly positive and encouraging, reflecting the workshop’s effectiveness in meeting objectives.

Participants expressed particular interest in the diverse panel, including a representative from a third-sector organization, an expert by experience, a carer, a psychiatrist and chaplaincy, thereby enriching the workshop’s relevance and depth. These findings indicate that the workshop effectively enhanced understanding of transcultural issues and resources, underscoring the significance of educational initiatives in fostering cultural competence within healthcare settings.

Organise further education opportunities. Include greater lived experience and scenario discussions in next workshops. Cover topics like old age psychiatry, cultural backgrounds, correct cultural terminologies and expression of mental distress. Disseminate data regionally and nationally to raise awareness.

